# Differences in concentration of neuron-specific enolase (NSE), neutrophil elastase (NE), and calcium-binding protein S100B in viral diseases: a pilot study focused on normoglycemic COVID-19 patients

**DOI:** 10.3389/fmolb.2026.1769050

**Published:** 2026-03-18

**Authors:** Joanna Adamiec-Mroczek, Agnieszka Bronowicka-Szydełko, Łukasz Lewandowski, Beata Nowak, Anna Turno-Kręcicka, Marta Misiuk-Hojło, Marta Stanek, Agnieszka Matera-Witkiewicz, Magdalena Krupińska, Kinga Gostomska-Pampuch, Edwin Kuźnik, Maciej Rabczyński, Małgorzata Matusiewicz, Izabela Berdowska, Martyna Korbecka, Daria Mykhailova, Zuzanna Przystupa, Beata Smyk, Anna Wojakowska, Amedeo Amedei, Małgorzata Trocha, Katarzyna Madziarska

**Affiliations:** 1 Clinical Department of Ophthalmology, Wroclaw Medical University, Wroclaw, Poland; 2 Department of Biochemistry and Immunochemistry, Wroclaw Medical University, Wroclaw, Poland; 3 Department of Physiology and Pathophysiology, Wroclaw Medical University, Wroclaw, Poland; 4 The Tadeusz Dorobisz Regional Centre for Blood Donation and Blood Treatment, Wroclaw, Poland; 5 Screening of Biological Activity Assays and Collection of Biological Material Laboratory, Wroclaw Medical University Biobank, Wroclaw, Poland; 6 Clinical Department of Diabetology, Hypertension and Internal Diseases, Institute of Internal Diseases, Wroclaw Medical University, Wroclaw, Poland; 7 Non-public Specialist Medical Practice Adrian Wojciech Przystupa, Bielsk Podlaski, Poland; 8 Department of Experimental and Clinical Medicine, University of Florence, Florence, Italy

**Keywords:** calcium-binding protein S100B, COVID-19, neuron-specific enolase (NSE), neutrophil elastase (NE), SARS-CoV-2

## Abstract

SARS-CoV-2 infection is characterized by a wide spectrum of clinical severity. Despite more than 2 years having passed since the end of the COVID-19 pandemic, the virus’s properties continue to form the basis for developing diagnostic and therapeutic models relevant to future epidemics. The aim of this study was to evaluate the diagnostic utility of classical inflammatory and metabolic markers, as well as potential variables associated with COVID-19 condition, such as neutrophil elastase (NE), neuron-specific enolase (NSE), and S100B protein. The analysis was conducted in carefully selected, homogeneous groups: the study group (patients with symptomatic COVID-19, without comorbidities) and the control group (healthy individuals, without comorbidities), with approximately 100 participants in each group. In the study group, significantly higher values were observed for numerous markers, including basophils, creatinine, eosinophils, HbA1c, HDL cholesterol, lymphocytes, monocytes, neutrophils, hemoglobin, hematocrit, platelets, as well as NSE and S100B. In a multivariate model assessing the likelihood of a result compared to the control group, monocyte count was positively associated with increased odds (OR = 1.6487; 95% CI: 1.2480–2.4318; p = 0.0001). Neutrophil count showed a positive association per 10-unit increase (OR = 1.0377; 95% CI: 1.0214–1.0622; p < 0.0001). Finally, NSE was also positively associated with increased odds (OR = 1.1466 per unit increase; 95% CI: 1.0175–1.2998; p = 0.0218).

## Graphical Abstract

**Figure:**
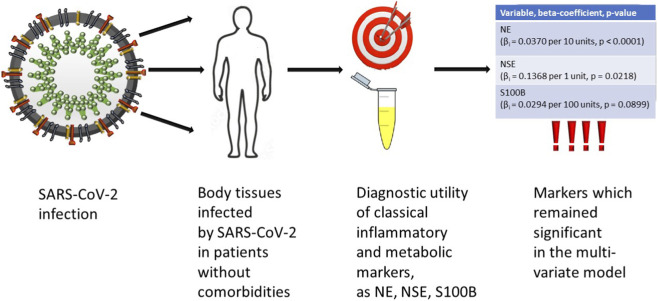

## 1 Introduction

The COVID-19 pandemic has demonstrated that the emergence of pathogens capable of causing life-threatening disease or substantially impairing the health of millions of people worldwide is a realistic scenario. This may result from multiple factors, including the intrinsic structure of the pathogen, which can confer specific biological properties such as multireceptor binding capacity. Intensive research conducted during the COVID-19 pandemic has shown that spike proteins of coronaviruses, including SARS-CoV-2, interact with several host cell proteins, such as angiotensin-converting enzyme 2 (ACE2) (Yan et al., 2020), basigin-2/EMMPRIN/CD147 (Cantuti-Castelvetri et al., 2020), and neuropilin-1 (NRP1) (Wang C. et al., 2020). These interactions may also involve cyclophilin A (CyPA), a CD147 ligand. The COVID-19 pandemic triggered a global, multifaceted crisis affecting, among others, the economy, tourism, education, and–most importantly–the healthcare sector (Boem et al., 2021). The rapid spread of infection necessitated the initiation of scientific efforts on a global scale aimed at developing vaccines and therapies designed to reduce the number of infections. Due to the structure and continuous mutations of SARS-CoV-2, as well as its multireceptor properties, new types of vaccines were developed and implemented, including mRNA, viral vector, protein-based, and DNA vaccines, offering a broad range of immunization strategies. In addition, these platforms may induce enhanced cellular and humoral immune responses, such as T helper (Th) cell responses, germinal center reactions, and the generation of appropriate memory cells (Li et al., 2022). Efforts have also been undertaken to develop new technologies, such as oral vaccines based on engineered microorganisms, nanoparticle delivery systems, self-amplifying RNA (saRNA), and artificial intelligence (AI)-assisted tools. Among these innovations, an oral probiotic antiviral platform based on *Escherichia coli* Nissle 1917 has been developed, capable of inducing both mucosal and systemic immunity (Kamble et al., 2025). This platform displays nanobodies targeting the spike protein on its surface or expresses the receptor-binding domain (RBD) of the spike protein. It has been demonstrated that EcN expressing nanobodies not only inhibits the interaction between spike-expressing pseudoviruses and the ACE2 receptor, but also enables the translocation of nanobodies to distant organs via outer membrane vesicles (OMVs). Thus, probiotic platforms may serve as the basis for developing an entirely new strategy to combat infections, based on the customization of biotherapeutics against various pathogens (Kamble et al., 2025).

Multireceptor binding increases viral infectivity by enabling multiple routes of cellular entry, more efficient replication, and a lower infectious dose (ID50). It also broadens viral tropism, allowing infection of different cell types within the same organ, multiple organs simultaneously, and even different host species. Furthermore, multireceptor capacity enhances virulence, facilitates immune evasion (e.g., through infection of immune cells leading to excessive inflammation, endothelial dysfunction, and coagulopathy), complicates therapeutic development (as blocking a single receptor is insufficient), and increases epidemic potential by promoting the emergence of more infectious variants. Consequently, these mechanisms contribute to a higher risk of cross-species transmission, more complex acute and post-acute clinical manifestations (including post-COVID-19 syndrome), chronic complications, prolonged recovery, and an increased need for long-term medical care.

During the early months of the COVID-19 pandemic, global mortality was estimated at approximately 3.68%, while mortality among critically ill patients reached up to 50% (Wang C. et al., 2020). These observations highlighted the importance of rapid clinical intervention and the urgent need to identify reliable predictors of severe disease. Numerous studies therefore focused on early diagnostic and prognostic biomarkers reflecting inflammatory and metabolic pathways. Commonly evaluated markers included D-dimer, procalcitonin, C-reactive protein (CRP), lymphocyte count (Jaskolowska et al., 2023), and red cell distribution width–standard deviation (RDW-SD), a hematological parameter reflecting disturbances in erythropoiesis, oxidative stress, and systemic inflammation–processes strongly associated with severe COVID-19 (Wang K. et al., 2020). Elevated RDW has been consistently associated with increased disease severity and mortality (Lee et al., 2021). A retrospective study of 592 patients demonstrated that high RDW at hospital admission was associated with increased mortality risk and could predict disease severity (Kouhpeikar et al., 2023). Similarly, patients with markedly elevated RDW experienced worse outcomes in both intensive care units and general medical wards (Moreno-Torres et al., 2022). In a cohort of 1,641 individuals, RDW-SD remained an independent prognostic marker after adjustment for age, CRP, lymphocyte count, and D-dimer levels (Foy et al., 2020). Importantly, dynamic changes in RDW were also clinically relevant, as increases in RDW-SD during hospitalization were strongly associated with clinical deterioration and higher mortality.

Despite these findings, severe COVID-19 can still occur among patients with elevated RDW-SD, indicating the need for additional prognostic markers, particularly those released into the extracellular space following tissue or organ damage. Potential candidates include neuron-specific enolase (NSE), neutrophil elastase (NE), and the calcium-binding protein S100B. Several studies have demonstrated that patients with severe COVID-19 exhibit significantly increased serum NE levels, which correlate with pro-inflammatory cytokines such as interleukin-6 (IL-6), tumor necrosis factor-α (TNF-α), and interleukin-8 (IL-8), and are associated with respiratory failure, poor clinical outcomes, and increased mortality (Guéant et al., 2021). NE has also been identified as an independent predictor of lung damage assessed by computed tomography (CT), the number of affected organs, and intensive care unit (ICU) admission (Guéant et al., 2021). In multivariate models adjusted for age, sex, and clinical prognostic variables, high NE concentrations (>150 ng/mL) independently predicted in-hospital mortality (Cardelli et al., 2022).

Neutrophil elastase is a key marker of neutrophil activation and degranulation, which are characteristic features of severe viral infections. NE plays a central role in the formation of neutrophil extracellular traps (NETs), DNA-based structures released by activated neutrophils. Patients with severe COVID-19 show increased circulating NETs and enhanced NE release, which may contribute to endothelial injury, microvascular thrombosis, and the development of acute respiratory distress syndrome (ARDS). By degrading elastin and extracellular matrix proteins, NE promotes alveolar epithelial damage, increases alveolar–capillary barrier permeability, and impairs gas exchange. Accordingly, elevated NE levels have been associated with more severe radiological lung changes and ARDS (Narasaraju et al., 2024). Moreover, NE correlates with CRP, D-dimer, ferritin, and IL-6 levels, indicating an increased risk of coagulopathy and thrombotic complications commonly observed in COVID-19.

S100B has also been reported to correlate with COVID-19 severity and with markers of systemic inflammation (ferritin, CRP, procalcitonin) and organ damage (alanine aminotransferase and creatinine), suggesting that S100B may reflect widespread inflammation and tissue injury (Aceti et al., 2020). However, other studies found no significant differences in S100B concentrations between COVID-19 patients and controls, observing associations mainly with neurological symptoms and only borderline statistical significance (Sahin et al., 2022). In contrast, elevated levels of S100B and NSE were reported in patients with severe COVID-19 admitted to the ICU, with S100B additionally associated with hypoperfusion markers and short-term mortality (Kokkoris et al., 2022). Increased serum NSE levels have also been observed in COVID-19 patients compared with controls, and NSE concentrations correlated with the extent of lung involvement (Cione et al., 2021). Patients with severe disease consistently exhibited higher NSE levels than those with mild or moderate COVID-19 (Sahin et al., 2023).

Importantly, most previous studies were conducted in older populations or in patients with multiple comorbidities. Data on individuals who experienced symptomatic COVID-19 without underlying chronic diseases remain limited. In such heterogeneous cohorts, the diagnostic value of individual biomarkers is difficult to interpret, as elevated levels may reflect pre-existing conditions rather than acute viral infection. The aim of the present study was to evaluate the diagnostic utility of established inflammatory and metabolic markers, as well as potential COVID-19–related biomarkers—NE, NSE, and S100B—in carefully selected, homogeneous study and control groups. Participants in both groups had no history of diabetes or neurological disorders, were aged 45–65 years, and were matched for group size (98 vs. 100 participants) and sex distribution. Multivariate logistic regression analysis was applied to identify patterns of simultaneous differences in multiple variables between COVID-19 patients and controls.

## 2 Materials and methods

A total of 275 serum samples were collected from patients with symptomatic COVID-19 treated at the Temporary Hospital in Wrocław (177 individuals) and from blood donors at the Regional Blood Donation and Blood Therapy Center named after Professor Tadeusz Dorobisz in Wrocław (98 individuals). The patients did not suffer from any other diseases that could significantly affect the obtained results. Individuals with psychiatric disorders, cancer, or diabetes were excluded from the study. The material obtained from patients was processed in the shortest possible time, i.e., within 1 hour of collection, properly secured, anonymized, and stored at −80 °C. The collection of biological material was approved by the Bioethics Committee of the Medical University of Wrocław (approval numbers KB 187/2019 and KB-666/2019). Blood samples from COVID-19 patients were collected at hospital admission (between November 2021 and February 2022), whereas blood from voluntary blood donors was collected in the autumn of 2019, before the pandemic. Therefore, it was certain that the control group had not been exposed to SARS-CoV-2.

The concentrations of NE, S100B, and NSE were determined according to the manufacturers’ protocols for the ELISA kits used (respectively: Human Neutrophil Elastase ELISA Kit, cat. no. E0778Hu, SUNLONG BIOTECH CO, Hangzhou, China; Human Protein S100-B ELISA Kit, cat. no. E2200HU, SUNLONG BIOTECH CO; and Human NSE (Neuron Specific Enolase) ELISA Kit, cat. no. DKO 073, DiaMetra, Spello, Italy). The assays used were characterized by high sensitivity and a broad range of detectable concentrations: for NE, sensitivity >31 pg/mL with a range of 78–5,000 pg/mL; for S100B, sensitivity >7.8 pg/mL with a range of 15.6–1,000 pg/mL; and for NSE, sensitivity >0.19 ng/mL with a range of 0.25–25 ng/mL. Absorbance values were read using a BioTek Synergy H1 Multimode Reader (Agilent, Santa Clara, CA, USA).

### 2.1 Data analysis strategy

Data analysis was performed in R (version 4.5.0) using packages including tidyverse, car, ResourceSelection, broom, pROC, and logistf. Key categorical variables (group, sexmale, s100b_positive) were converted to factors. To reduce biological heterogeneity related to chronic hyperglycemia, we applied an a priori exclusion of participants with HbA1c ≥ 6.5%. This threshold reflects the standard diagnostic cut-off for diabetes and was used to avoid confounding of S100B, NSE and immune-cell parameters by underlying hyperglycemia, which can independently alter these biomarkers. The intention was to isolate inflammation-driven alterations (e.g., viral or immunologic) from metabolic effects, thereby ensuring a more coherent biological signal for the regression analyses. Data integrity checks confirmed a binary group variable suitable for stratified analysis. Univariate analysis for continuous variables was conducted using Wilcoxon rank-sum tests (U test), reporting medians, interquartile ranges (Q1, Q3), minimums, and maximums. Categorical variables were compared with chi-squared or Fisher’s exact tests when expected counts were low. Linearity of continuous variables (NE, NSE, S100B) on the logit scale was assessed via the Box-Tidwell test on standardized variables. Although the test suggested potential nonlinearity for ne (p = 0.017) and nse (p = 0.0003), convergence warnings and quasi-complete separation issues led to discarding nonlinear terms in favor of a simpler linear logistic model to preserve stability and interpretability. Continuous variables were median-centered, with additional scaling for ne and s100b to improve interpretability. Logistic regression incorporating these transformed variables and sexmale was fit, followed by multicollinearity assessment via Variance Inflation Factor (VIF). All VIF values were well below critical thresholds, with the highest for NE (2.24), MON (2.04), and HDL (2.04), indicating acceptable variable independence. Model fit was evaluated using the Hosmer-Lemeshow goodness-of-fit test (χ^2^ = 11.29, df = 8, p = 0.186), supporting adequate model calibration. Given satisfactory multicollinearity and model fit diagnostics, a Firth-penalized logistic regression was applied to address potential bias from data separation and improve estimate stability, confirming robustness consistent with the classical model. All regression analyses were conducted on a complete-case analytic sample, defined strictly by non-missing values across all model variables. This combined approach of rigorous preprocessing, assumption verification, and penalized regression modeling ensured a stable and interpretable framework for subsequent inference.

## 3 Results

### 3.1 Univariate characteristics of the population sample

The study included 198 participants, divided nearly equally into control (n = 98) and case (n = 100) groups. Key demographic and clinical features were compared between groups (Table 1). Supplementary Material (Supplementary Table S1) features information on the complete case dataset which was used for multivariate modeling.

**TABLE 1:** Characteristics of the population sample revealing univariate differences between the two groups (based the complete dataset).

Quantitative features													
Feature	Control (N = 98)						Case (N = 100)						p
	Min	Q1	Median	Q3	Max	% Missing	Min	Q1	Median	Q3	Max	% Missing	
Age	46.00	49.00	51.00	55.00	73.00	0.00	27.00	43.25	57.50	69.00	91.00	2.00	0.0847
ALP						100.00	19.00	52.50	65.00	84.00	250.00	5.00	
BAS	0.10	0.30	0.40	0.50	0.80	0.00	0.00	0.10	0.20	0.30	1.40	1.00	**<0.0001**
Bicarbonate						100.00	22.80	26.10	27.30	29.30	34.90	59.00	
Clac						100.00	0.70	1.00	1.20	1.40	2.60	59.00	
Creatinine	0.59	0.93	1.03	1.15	1.64	0.00	0.43	0.74	0.81	0.94	1.47	36.00	**<0.0001**
CtO2						100.00	3.50	8.10	14.10	18.20	24.10	60.00	
D-dimers						100.00	0.28	0.58	0.81	1.17	88.26	1.00	
eGFR						100.00	44.00	76.75	94.00	108.00	152.00	36.00	
EOS	1.10	2.10	3.00	3.75	7.70	0.00	0.00	0.00	0.00	0.33	4.10	0.00	**<0.0001**
Ferritin						100.00	26.70	351.05	717.40	1191.53	5,550.00	2.00	
Ft3						100.00	1.06	1.48	1.90	2.03	2.62	71.00	
Ft4						100.00	0.72	0.91	1.00	1.11	1.74	6.00	
Ggtp						100.00	9.00	33.00	50.00	118.50	818.00	1.00	
Glucose						100.00	63.00	88.25	99.50	124.75	203.00	2.00	
HbA1c	4.90	5.30	5.40	5.60	6.20	0.00	4.90	5.60	5.80	6.10	6.40	0.00	**<0.0001**
HCT	38.00	42.03	44.00	45.68	49.70	0.00	30.60	37.93	40.85	43.23	51.90	0.00	**<0.0001**
HDL	24.50	41.43	51.85	61.23	95.30	0.00	11.00	30.00	36.00	42.50	88.00	8.00	**<0.0001**
HGB	12.40	14.13	14.90	15.50	17.00	0.00	10.50	12.58	13.60	14.53	17.90	0.00	**<0.0001**
hsCRP						100.00	0.94	25.60	46.22	89.90	230.06	0.00	
IL6						100.00	2.00	4.79	11.90	29.90	6120.00	7.00	
INR						100.00	0.88	1.03	1.09	1.17	4.39	4.00	
LDH						100.00	138.00	261.00	364.50	442.50	1052.00	2.00	
LDLC	24.02	97.62	122.66	143.46	211.44	4.08	28.00	71.75	92.00	110.50	152.00	8.00	**<0.0001**
LYM	20.70	28.05	32.55	36.48	47.80	0.00	3.50	13.20	19.50	26.35	52.70	0.00	**<0.0001**
MCHC	31.80	33.40	33.80	34.10	35.00	0.00	31.20	32.81	33.40	34.05	36.40	10.00	**0.0028**
MCV	78.00	88.00	91.50	95.00	100.00	0.00	79.70	86.90	89.65	92.10	106.70	0.00	**0.0162**
MON	3.80	5.33	6.35	7.50	12.30	0.00	2.30	5.85	8.35	10.98	29.80	0.00	**0.0001**
MOSM						100.00	237.70	284.70	288.60	293.40	306.70	59.00	
MPV	6.80	7.63	7.90	8.60	9.60	0.00	9.00	9.89	10.30	11.10	13.85	10.00	**<0.0001**
NE	0.00	221.75	457.80	591.15	2045.00	10.20	297.00	952.50	1160.00	1441.75	4165.00	0.00	**<0.0001**
NEU	42.70	52.38	57.35	61.65	72.80	0.00	34.90	62.38	71.65	79.00	89.20	0.00	**<0.0001**
NSE	4.59	6.90	8.63	10.93	38.38	10.20	4.08	10.39	13.81	19.42	64.83	0.00	**<0.0001**
NTPROBNP						100.00	8.30	63.00	127.70	321.50	4739.30	19.00	
pCO2						100.00	29.30	35.20	40.60	43.70	60.40	59.00	
PCT						100.00	0.01	0.03	0.06	0.09	0.48	0.00	
PH						100.00	7.36	7.41	7.43	7.48	7.55	59.00	
PLT	159.00	213.00	244.00	277.50	367.00	0.00	2.00	153.50	205.00	314.00	654.00	0.00	**0.0162**
PO2						100.00	15.00	23.90	33.90	59.70	121.00	59.00	
Potassium						100.00	2.63	3.91	4.23	4.54	157.12	0.00	
PT						100.00	10.20	11.98	12.70	13.63	50.90	4.00	
RBC	3.99	4.56	4.83	5.08	5.68	0.00	3.33	4.17	4.55	4.82	6.22	0.00	**<0.0001**
RDWCV	36.00	40.00	42.00	43.00	52.00	0.00	34.40	40.26	42.58	45.25	65.65	10.00	0.0717
RDWSD	11.40	12.30	12.90	13.80	17.50	0.00	11.60	12.46	12.89	13.60	17.88	10.00	0.9092
S100B	0.00	0.00	0.00	0.00	11270.00	8.16	555.94	777.45	928.75	1078.35	8095.65	1.00	**<0.0001**
SO2						100.00	16.80	40.40	72.00	90.80	99.20	59.00	
Sodium						100.00	116.00	137.00	139.00	142.00	152.00	2.00	
Stand_bicarbonate						100.00	23.50	25.30	26.20	28.30	33.10	59.00	
TCHOL	143.00	186.50	212.00	238.00	300.00	0.00	54.00	132.50	153.00	184.00	245.00	8.00	**<0.0001**
TG	47.00	132.00	171.00	224.25	1350.00	0.00	46.00	92.50	118.50	154.50	303.00	8.00	**<0.0001**
TPROT	6.40	7.10	7.40	7.70	213.00	0.00	5.40	5.90	6.20	6.60	7.60	67.00	**<0.0001**
TSH						100.00	0.08	0.53	0.85	1.24	4.19	3.00	
UREA						100.00	10.00	27.00	32.00	40.00	170.00	0.00	
URICACID						100.00	2.00	3.50	4.40	5.23	12.50	4.00	
WBC	3.80	5.40	6.45	7.40	10.10	0.00	1.69	3.93	5.60	6.98	20.69	5.00	**0.0014**

Qualitative features					
Feature	Category	Group	n	%	p
S100B positive	No	Control	96	99	**<0.0001**
S100B positive	No	Case	1	1	
S100B positive	Yes	Control	2	2	
S100B positive	Yes	Case	99	98	
Sexmale	No	Control	28	43.1	0.2664
Sexmale	No	Case	37	56.9	
Sexmale	Yes	Control	70	52.6	
Sexmale	Yes	Case	63	47.4	

Statistically significant values have been highlighted in bold.

No significant difference in age was observed between groups (median 51 vs. 57.5 years, p = 0.085). However, multiple biomarkers showed significant between-group differences (p < 0.001) between cases and controls, mirroring distinct biological profiles. For instance, median values of inflammatory and metabolic markers such as basophils (0.40 vs. 0.20, p < 0.0001), creatinine (1.03 vs. 0.81, p < 0.0001), eosinophils (3.00 vs. 0.00, p < 0.0001), Hba1c (5.40 vs. 5.80, p < 0.0001), HDL cholesterol (51.85 vs. 36.00, p < 0.0001), lymphocytes (32.55 vs. 19.50, p < 0.0001), monocytes (6.35 vs. 8.35, p = 0.0001), neutrophils (57.35 vs. 71.65, p < 0.0001), NSE (8.63 vs. 13.81, p < 0.0001), and S100b protein (median 0 vs. 928.75, p < 0.0001) were markedly different, indicating pronounced immunologic and biochemical alterations in the case group.

Other hematological indices such as hemoglobin (14.90 vs. 13.60, p < 0.0001), hematocrit (44.00 vs. 40.85, p < 0.0001), and platelet count (244.00 vs. 205.00, p = 0.0162) also differed significantly.

Several variables had substantial missingness (e.g., bicarbonate, chloride, ferritin), limiting interpretation for those markers.

The distribution of sex (male) did not significantly differ between groups (p = 0.27), with approximately equal proportions in controls and cases. In contrast, S100B positivity was almost exclusively present in cases (98%) versus controls (2%), reaching clear statistical significance (p < 0.0001).

### 3.2 Additional clinical characteristics of the study group

Data completeness varied across variables and is reported explicitly to allow transparent interpretation of the presented summaries (Table 2).

**TABLE 2:** Additional clinical characteristics of the study group.

Variable	N	n non-missing	n missing	% Missing	Summary
Hospitalization outcome	100	93	7	7.0%	Discharge home: 89 (95.7%) | acute transfer: 4 (4.3%) | Rehabilitation/further treatment: 0 (0.0%) | death: 0 (0.0%)
Admission SBP [mmHg]	100	94	6	6.0%	130.00 [120.00–143.00]
Admission DBP [mmHg]	100	94	6	6.0%	79.50 [70.00–86.00]
Admission HR [bpm]	100	94	6	6.0%	86.00 [80.00–96.00]
Admission (room air) SpO_2_ [%]	100	67	33	33.0%	93.00 [88.00–96.00]
Admission (oxygen therapy) SpO_2_ [%]	100	61	39	39.0%	97.00 [95.00–98.00]
Respiratory support (admission)	100	90	10	10.0%	No oxygen: 33 (36.7%) | Low-flow oxygen: 57 (63.3%) | High-flow oxygen (HFNC): 0 (0.0%) | Non-invasive ventilation (NIV): 0 (0.0%) | Invasive mechanical ventilation (IMV): 0 (0.0%)
Oxygenation status (admission)^a^	100	94	6	6.0%	High: 27 (28.7%) | moderate: 47 (50.0%) | low: 20 (21.3%)
Consciousness (admission)	100	94	6	6.0%	Alert: 93 (98.9%) | Somnolent/confused: 1 (1.1%) | Unconscious (not intubated): 0 (0.0%) | Unconscious (intubated): 0 (0.0%)
GCS score (admission)	100	17	83	83.0%	15.00 [15.00–15.00]
Body temperature (admission) [°C]	100	87	13	13.0%	36.70 [36.40–37.40]
Pulmonary congestion (admission)	100	62	38	38.0%	No: 61 (98.4%) | Yes: 1 (1.6%)
Crackles (admission)	100	73	27	27.0%	No: 36 (49.3%) | Yes: 37 (50.7%)
Wheezing (admission)	100	71	29	29.0%	No: 53 (74.6%) | Yes: 18 (25.4%)
Dyspnea (admission)	100	87	13	13.0%	No: 18 (20.7%) | Yes: 69 (79.3%)
Chest pain (admission)	100	63	37	37.0%	No: 50 (79.4%) | Yes: 13 (20.6%)
Cough (admission)	100	87	13	13.0%	No: 19 (21.8%) | Yes: 68 (78.2%)
Hemoptysis (admission)	100	62	38	38.0%	No: 57 (91.9%) | Yes: 5 (8.1%)
Anosmia (admission)	100	64	36	36.0%	No: 53 (82.8%) | Yes: 11 (17.2%)
Ageusia (admission)	100	64	36	36.0%	No: 54 (84.4%) | Yes: 10 (15.6%)

Quantitative variables are summarized as median [first quartile–third quartile], while qualitative — as count (frequency); for qualitative variables, the categories with no patients are kept in the summary, for clarity.

^a^ oxygenation status (admission) was defined as: High — SpO_2_ ≥95% on room air, or (if room-air SpO_2_ missing) no oxygen supplementation at admission; moderate — SpO_2_ 90%–94% on room air, or (if room-air SpO_2_ missing) low-flow oxygen therapy at admission; low — SpO_2_ <90% on room air; patients with missing data on both room-air SpO_2_ and respiratory support were not classified.

Regarding hospitalization outcomes, information was available for 93 patients. The huge majority were discharged home (89/93, 95.7%), while acute transfer to another hospital occurred in 4 patients (4.3%). No in-hospital deaths or transfers for rehabilitation or further treatment were recorded in this dataset.

At admission, patients were hemodynamically stable. Median systolic blood pressure was 130 mmHg [120–143], diastolic blood pressure 79.5 mmHg [70–86], and heart rate 86 bpm [80–96], with approximately 6% missing data for these parameters. Median body temperature at admission was 36.7 °C [36.4–37.4], based on data available for 87 patients.

Oxygenation markers at admission were partly reported. Peripheral oxygen saturation measured on room air was available in 67% of patients and had a median of 93% [88–96], while saturation under oxygen therapy was recorded in 61% of patients, with a median of 97% [95–98]. With respect to respiratory support, data were available for 90 patients: 36.7% required no oxygen supplementation and 63.3% received low-flow oxygen therapy. No patients required high-flow oxygen therapy, non-invasive ventilation, or invasive mechanical ventilation at admission.

To provide a structured oxygenation description at presentation using the available retrospective data, an exploratory oxygenation status (admission) variable was derived based on room-air SpO_2_ thresholds and, when missing, on respiratory support at admission (see raw data: http://dx.doi.org/10.60956/11k6-bh03 for definitions). Using this derived variable, half of the patients were classified as having moderate oxygenation status at admission (50.0%), while 28.7% had high and 21.3% had low oxygenation status. This distribution is consistent with a cohort without advanced respiratory support requirements, but with frequent disturbances in oxygenation at admission.

Assessment of neurological status showed that almost all patients with available data were alert at admission (93/94, 98.9%), with only one patient classified as somnolent or confused. The Glasgow Coma Scale score was reported in a limited subset of patients (17%), and when documented, was uniformly 15 points.

Admission symptoms were largely respiratory in nature. Dyspnea and cough were frequently reported, present in 79.3% and 78.2% of patients with available data, respectively. Crackles on auscultation were observed in approximately half of assessed patients, while wheezing was less common (25.4%). Chest pain was reported by 20.6% of patients, and hemoptysis was rare (8.1%). Disturbances of smell and taste were documented in a minority of patients, with anosmia present in 17.2% and ageusia in 15.6% of those assessed. Signs of pulmonary congestion at admission were uncommon (1.6%).

Overall, the study cohort consisted predominantly of patients presenting with respiratory symptoms and preserved hemodynamic stability, while the documentation completeness varied across clinical domains, mainly for oxygenation measures and neurological scoring.

### 3.3 Multivariate analysis–assessing the association of NE, NSE, and S100B with the odds of being in the case group (vs. control)

Firth-penalized logistic regression was applied to decrease bias due to data separation and to stabilize parameter estimates (Table 3).

**TABLE 3:** Firth penalized logistic regression model investigating the association of the clinical features/markers with the odds of being in the study group (COVID-19).

Feature	Interpretation	βi	βi SE	χ2	p	OR	Or -95% CI	Or 95% CI
(Intercept)	The odds of being in the study group given female sex and median values of the following features: Age (52), hba1c (5.60), hdl-c (41.40), lymph (27.80), mono (7.00), ne (842.30), nse (10.89), and s100b (684.13)	1.5161	0.7017	4.1872	**0.0407**	4.5543	1.0638	25.5385
Sexmale1	Fold difference in odds between males and females	−2.3327	0.8780	6.8233	**0.0090**	0.0970	0.0110	0.5756
age_med52.00	Fold difference in odds with a 1-year increase in age	−0.0027	0.0340	0.0045	0.9463	0.9973	0.9121	1.0793
hba1c_med5.60	Fold difference in odds with a one-unit increase in hba1c	2.1748	1.0228	3.8339	0.0502	8.8002	0.9979	116.6256
hdl_med41.40	Fold difference in odds with a one-unit increase in hdl-c	−0.1164	0.0331	12.7557	**0.0004**	0.8901	0.8147	0.9536
lym_med27.80	Fold difference in odds with a one-unit increase in lymph	−0.1433	0.0455	11.4988	**0.0007**	0.8665	0.7675	0.9462
Mon_med7.00	Fold difference in odds with a one-unit increase in mono	0.5000	0.1428	15.4149	**0.0001**	1.6487	1.2480	2.4318
ne_med842.30_per10units	Fold difference in odds with a 10-unit increase in ne	0.0370	0.0083	28.1760	**<0.0001**	1.0377	1.0214	1.0622
nse_med10.89	Fold difference in odds with a one-unit increase in nse	0.1368	0.0514	5.2644	**0.0218**	1.1466	1.0175	1.2998
s100b_med684.13_per100units	Fold difference in odds with a 100-unit increase in s100b	0.0294	0.0160	2.8761	0.0899	1.0298	0.9946	1.0668

Statistically significant values have been highlighted in bold.

**FIGURE 1:**
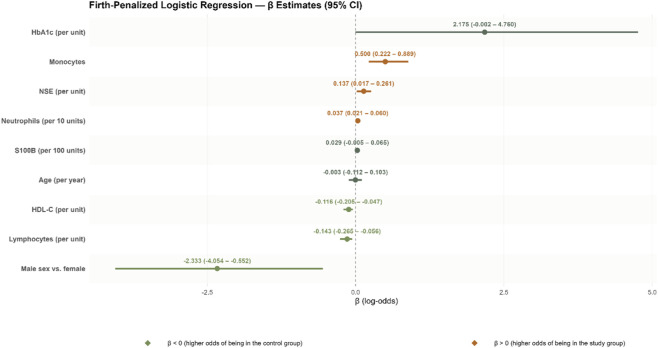
Forest plot featuring odds ratios (OR) of being in the study group vs. control according to the Firth penalized multivariate logistic regression model shown in Table 3.

The overall model was highly significant (Likelihood ratio test: χ^2^ (9) = 176.0100, p < 0.0010). Male sex was associated with markedly lower odds of the outcome (OR = 0.0970; 95% CI: 0.0110–0.5756; p = 0.0090). HbA1c demonstrated a borderline positive association (OR = 8.8002; 95% CI: 0.9979–116.6256; p = 0.0502), although the wide confidence interval indicates substantial uncertainty around the exact effect size. HDL cholesterol was inversely related to the outcome (OR = 0.8901 per 1 mg/dL increase; 95% CI: 0.8147–0.9536; p = 0.0004), while lymphocyte count also showed a protective effect (OR = 0.8665; 95% CI: 0.7675–0.9462; p = 0.0007). Conversely, monocyte count was positively associated with increased odds (OR = 1.6487; 95% CI: 1.2480–2.4318; p = 0.0001). Neutrophil count exhibited a strong positive relationship per 10-unit increase (OR = 1.0377; 95% CI: 1.0214–1.0622; p < 0.0001). Neuron-specific enolase (NSE) was similarly positively associated (OR = 1.1466 per unit increase; 95% CI: 1.0175–1.2998; p = 0.0218). S100B showed a modest, statistically non-significant tendency toward higher odds (OR = 1.0298 per 100 units; 95% CI: 0.9946–1.0668; p = 0.0899). Age was not significantly associated with the outcome (OR = 0.9973; 95% CI: 0.9121–1.0793; p = 0.9463). These results highlight the critical relevance of inflammatory and biochemical markers, along with sex differences, in influencing the studied outcome. The Firth correction ensured robust, reliable estimates despite potential data separation. The estimates are visualized in Figure 1.

## 4 Discussion

NE, S100B, and NSE are proteins that have demonstrated potential diagnostic and prognostic value in patients with COVID-19. Previous studies have shown, among other findings, that NE levels are significantly associated with an increased risk of in-hospital mortality (Cardelli et al., 2022). Serum S100B levels in hospitalized COVID-19 patients have been reported to correlate with disease severity, with higher concentrations associated with a more severe clinical course, including increased intensity of care and higher COVID severity scores (Aceti et al., 2020). NSE levels, in turn, have been linked to lung injury—patients with dyspnea exhibited higher NSE concentrations (Cione et al., 2021)—as well as to subclinical brain damage caused by SARS-CoV-2 infection (Silva et al., 2023). These studies were primarily conducted in hospitalized patients with fully symptomatic COVID-19 and without detailed stratification according to comorbidities. This raises the question of whether these proteins may have diagnostic value in patients hospitalized with severe COVID-19 who do not have comorbidities such as diabetes, neurological, autoimmune, or oncological diseases, and in whom disease severity is difficult to predict.

In the present study, we evaluated selected biological markers (NE, NSE, and S100B) in the context of SARS-CoV-2 infection. To date, most studies have focused on patients with severe disease or significant comorbidities. For example, in patients with type 2 diabetes following COVID-19, significant increases in NE, NSE, and S100B levels were observed, particularly among those with diabetic nephropathy (Rabczyński et al.). In contrast, although our study included patients in the acute phase of symptomatic COVID-19, none had diabetes or other chronic comorbidities, including neurological, autoimmune, or oncological conditions. Selected clinical parameters in healthy volunteers and SARS-CoV-2–infected patients–including blood gas measurements, coagulation parameters, kidney function indices, electrolytes, and standard inflammatory markers such as CRP and IL-6 – did not differ significantly between groups. This finding confirms the absence of comorbidities known to increase baseline IL-6 production, such as obesity, diabetes, or chronic inflammatory diseases (Ellulu et al., 2017). Additionally, no statistically significant differences were observed in RDW-SD values, although an observational study of 1,641 patients previously demonstrated an association between elevated RDW and COVID-19 severity (Foy et al., 2020).

Given the lack of association between severe COVID-19 and standard indicators of inflammation, immune dysregulation, or erythropoiesis in our cohort, we investigated the diagnostic utility of NSE, S100B, and NE. Enolases are enzymes essential for cellular energy metabolism, functioning in the glycolytic pathway. NSE is a γ-subunit–containing enolase predominantly expressed in neurons and neuroendocrine cells and is released into the circulation following neuronal injury, damage to neuroendocrine cells, hypoxia, systemic stress, or intense inflammatory responses. Elevated serum NSE levels have been reported in conditions such as stroke, ischemia–reperfusion brain injury, and hypertension (Dagonnier et al., 2021; Bharosay et al., 2018; Iłżecki et al., 2016). Other reports suggest that NSE may serve as a marker of neurological damage associated with COVID-19, particularly in patients presenting with symptoms such as altered consciousness, confusion, or memory impairment (Alvarez et al., 2022). Elevated NSE levels have also been associated with chronic fatigue syndrome-like symptoms and affective disturbances related to long COVID-19 (Al Masoodi et al., 2025).

In our study, NSE concentrations were significantly higher in hospitalized COVID-19 patients compared with healthy controls. Importantly, these patients did not exhibit overt neurological symptoms at hospital admission. Neurological assessment showed that almost all patients with available data were alert at admission (93/94, 98.9%), with only one patient classified as somnolent or confused. The Glasgow Coma Scale score was reported in a limited subset of patients (17%), and in all documented cases it was 15 points. These findings suggest that NSE may serve as a marker of acute SARS-CoV-2 infection even in the absence of clinically apparent neurological deficits. Furthermore, Firth logistic regression analysis demonstrated that higher NSE concentrations were associated with an increased likelihood of belonging to the COVID-19 patient group.

NE plays a key role in immune system activation and can contribute to tissue damage, particularly in the lungs, thereby promoting the development of acute respiratory distress syndrome (ARDS) (Zuo et al., 2020). On the one hand, NE has been shown to activate the coronavirus spike protein, altering viral conformation and facilitating infection of human cells (Belouzard et al., 2010). On the other hand, NE released from neutrophil granules participates in inflammatory responses and in the formation of neutrophil extracellular traps (NETs) – web-like structures composed of chromatin and granule-derived proteins (Pisani et al., 2025). NE-DNA complexes serve as indicators of neutrophil hyperactivity and immunothrombogenesis (Papayannopoulos, 2018). Elevated NE levels are associated with worse clinical outcomes and may have prognostic significance in hospitalized patients (Zuo et al., 2020). The marked increase in NE observed in COVID-19 patients in our study supports its potential role as both a prognostic marker and a therapeutic target, as previously suggested by Guéant et al. (2021).

S100B is a marker of blood–brain barrier (BBB) disruption and is considered useful in assessing the severity of neurological complications. Positive correlations between S100B levels and serum IL-6 concentrations, lymphopenia, hypoperfusion markers, disease severity, and short-term mortality have been reported (Kokkoris et al., 2022). Bisulli et al. (2025) proposed the use of S100B as a predictor of adverse outcomes in COVID-19. In our study, S100B concentrations were higher in COVID-19 patients than in controls, although statistical significance was not reached in the Firth regression model. Nevertheless, positive S100B values were observed almost exclusively in COVID-19 patients.

Elevated levels of NE, NSE, and S100B may reflect complex pathophysiological mechanisms underlying COVID-19, including immune activation, oxidative stress, neuronal injury, and neuroinflammatory responses. Additionally, significant intergroup differences were observed in kidney function parameters, lipid profiles, glycated hemoglobin (HbA1c), and blood morphology. Typical COVID-19–associated alterations in the white blood cell profile, such as leukocytosis, neutrophilia, and lymphopenia, have been linked to poorer prognosis (Qin et al., 2020; Liu et al., 2020). Similar associations have been reported for thrombocytopenia (Lippi et al., 2020). In our cohort, platelet counts remained within normal reference ranges but differed significantly between groups.

Numerous studies have reported impaired kidney function in COVID-19 patients, particularly in severe cases (Rabczyński et al., 2024; Su et al., 2020; Cheng et al., 2020). Interestingly, the lower creatinine levels and higher estimated glomerular filtration rate (eGFR) observed in our study may indicate transient hyperfiltration during acute inflammatory states. We also observed significant reductions in lipid profile parameters–including total cholesterol (TC), low-density lipoprotein (LDL), high-density lipoprotein (HDL), and triglycerides (TG) – consistent with previous reports and likely related to cytokine activation and systemic inflammation during SARS-CoV-2 infection (Wei et al., 2020; Fan et al., 2020). Lipid profile assessment may therefore represent a simple and accessible tool for COVID-19 risk evaluation. Similarly, HbA1c, reflecting glycemic control over the preceding 3 months, may serve as a relevant prognostic indicator. Higher HbA1c levels have been associated with increased susceptibility to severe infections, a higher risk of pneumonia, and renal complications (Zhu et al., 2020; Sardu et al., 2020). Notably, despite excluding patients with HbA1c values diagnostic of diabetes, significantly higher HbA1c levels were observed in COVID-19 patients in our study.

## 5 Conclusion

At the univariate level, NE, NSE and S100B concentrations were markedly higher in the case group. However, feature selection for multivariable modeling was not performed by forced inclusion; instead, we applied L1-penalized logistic regression (lasso; α = 1) to identify a parsimonious subset of variables with stable empirical signal. The lasso-selected variables were subsequently refit using Firth-penalized logistic regression to mitigate small-sample bias and separation. Within this framework, NE and NSE retained statistically robust associations with case status in the adjusted Firth model, documenting that their univariate contrasts persisted after penalization and refitting. In contrast, the strong univariate difference in S100B did not translate into an independent association, plausibly mirroring shared variance with other inflammatory markers. Additional laboratory differences–including lipid fractions, creatinine, lymphocyte and monocyte counts, hemoglobin parameters–showed large univariate contrasts but did not remain selected after L1-penalization, suggesting that these signals reflect the global metabolic and hematologic perturbation characteristic of acute infection rather than autonomous associations.

Given the cross-sectional design, the pronounced clinical asymmetry between groups, and the penalized feature-selection strategy, these findings must not be interpreted in causal, diagnostic or prognostic terms. The associations reported here represent patterns of co-occurrence in this specific sample structure. Within these constraints, NE and NSE emerge as the most consistent markers distinguishing case from control status after penalization and model stabilization.

The obtained results are descriptive and associative rather than prognostic. Overall, the data delineate the biochemical and immunologic profile of acutely hospitalized COVID-19 patients relative to a metabolically homogeneous control cohort, offering a cautious descriptive map of the characteristic alterations of acute systemic infection.

## 6 Limitations

The main limitation of our study seems to be its cross-sectional nature of the study, in which we analyzed selected parameters at a single point in time, and do not have information about the dynamics of changes in these parameters over time. In addition, we are unable to perform a retrospective classification of the patient group according to COVID-19 disease severity based on the classifications defined by the World Health Organization (WHO), the National Institutes of Health (NIH), or the European Centre for Disease Prevention and Control (ECDC) for the information lacks on respiratory disorders such as acute respiratory distress syndrome (ARDS).

## Data Availability

The original contributions presented in the study are publicly available. This data can be found here: http://dx.doi.org/10.60956/11k6-bh03.
