# Supplementary material for: Differences in concentration of neuron-specific enolase (NSE), neutrophil elastase (NE), and calcium-binding protein S100B in viral diseases: a pilot study focused on normoglycemic COVID-19 patients
Source: Front Mol Biosci. 2026 Mar 18;13:1769050. doi: 10.3389/fmolb.2026.1769050 (PMC13038554; doi:10.3389/fmolb.2026.1769050)
Supplement: Supplementary file 1 [file Table1.docx]

**Supplementary Materials**

**Table S1.** Characteristics of the population sample revealing univariate differences between the two groups (based on the complete-case analytic dataset)

| **Quantitative features** | | | | | | | | | | | | | | | |
| --- | --- | --- | --- | --- | --- | --- | --- | --- | --- | --- | --- | --- | --- | --- | --- |
| **feature** | **control (N = 79)** | | | | | | | **case (N = 90)** | | | | | | | **p** |
|  | **n** | **min** | **Q1** | **median** | **Q3** | **max** | **% missing** | **n** | **min** | **Q1** | **median** | **Q3** | **max** | **% missing** |  |
| Age | 79 | 46.00 | 49.00 | 51.00 | 55.00 | 73.00 | 0.00 | 90 | 27 | 43.25 | 57.00 | 67.75 | 91.00 | 0.00 | 0.1404 |
| ALP | 0 |  |  |  |  |  | 100.00 | 89 | 19 | 53.00 | 65.00 | 84.00 | 250.00 | 1.11 |  |
| BAS | 79 | 0.10 | 0.30 | 0.40 | 0.50 | 0.80 | 0.00 | 89 | 0 | 0.10 | 0.20 | 0.30 | 1.40 | 1.11 | **<0.0001** |
| BICARBONATE | 0 |  |  |  |  |  | 100.00 | 35 | 23 | 26.15 | 27.30 | 29.20 | 34.90 | 61.11 |  |
| CLAC | 0 |  |  |  |  |  | 100.00 | 35 | 1 | 0.95 | 1.20 | 1.40 | 2.60 | 61.11 |  |
| CREATININE | 79 | 0.62 | 0.94 | 1.03 | 1.15 | 1.64 | 0.00 | 58 | 0 | 0.74 | 0.81 | 0.94 | 1.47 | 35.56 | **<0.0001** |
| CTO2 | 0 |  |  |  |  |  | 100.00 | 34 | 4 | 7.65 | 13.30 | 18.60 | 24.10 | 62.22 |  |
| DDIMERS | 0 |  |  |  |  |  | 100.00 | 90 | 0 | 0.58 | 0.75 | 1.18 | 88.26 | 0.00 |  |
| EGFR | 0 |  |  |  |  |  | 100.00 | 58 | 44 | 77.75 | 94.00 | 107.75 | 152.00 | 35.56 |  |
| EOS | 79 | 1.10 | 2.15 | 3.00 | 3.85 | 7.70 | 0.00 | 90 | 0 | 0.00 | 0.00 | 0.40 | 4.10 | 0.00 | **<0.0001** |
| FERRITIN | 0 |  |  |  |  |  | 100.00 | 90 | 27 | 351.05 | 717.40 | 1191.53 | 5550.00 | 0.00 |  |
| FT3 | 0 |  |  |  |  |  | 100.00 | 27 | 1 | 1.53 | 1.90 | 2.07 | 2.62 | 70.00 |  |
| FT4 | 0 |  |  |  |  |  | 100.00 | 90 | 1 | 0.92 | 1.00 | 1.10 | 1.74 | 0.00 |  |
| GGTP | 0 |  |  |  |  |  | 100.00 | 90 | 9 | 31.25 | 49.00 | 111.50 | 818.00 | 0.00 |  |
| GLUCOSE | 0 |  |  |  |  |  | 100.00 | 89 | 63 | 87.00 | 99.00 | 124.00 | 203.00 | 1.11 |  |
| HbA1c | 79 | 4.90 | 5.20 | 5.40 | 5.60 | 6.10 | 0.00 | 90 | 5 | 5.60 | 5.90 | 6.10 | 6.40 | 0.00 | **<0.0001** |
| HCT | 79 | 38.00 | 42.05 | 43.90 | 45.75 | 49.70 | 0.00 | 90 | 31 | 38.08 | 40.85 | 43.45 | 51.90 | 0.00 | **<0.0001** |
| HDL | 79 | 24.50 | 41.15 | 51.30 | 60.00 | 95.30 | 0.00 | 90 | 11 | 30.00 | 36.00 | 42.00 | 88.00 | 0.00 | **<0.0001** |
| HGB | 79 | 12.40 | 14.10 | 14.80 | 15.50 | 17.00 | 0.00 | 90 | 11 | 12.60 | 13.60 | 14.58 | 17.90 | 0.00 | **<0.0001** |
| HSCRP | 0 |  |  |  |  |  | 100.00 | 90 | 1 | 24.92 | 46.69 | 89.63 | 205.45 | 0.00 |  |
| IL6 | 0 |  |  |  |  |  | 100.00 | 84 | 2 | 4.66 | 11.75 | 30.23 | 6104.00 | 6.67 |  |
| INR | 0 |  |  |  |  |  | 100.00 | 87 | 1 | 1.03 | 1.09 | 1.17 | 4.39 | 3.33 |  |
| LDH | 0 |  |  |  |  |  | 100.00 | 90 | 138 | 261.00 | 366.00 | 442.50 | 1052.00 | 0.00 |  |
| LDLC | 75 | 24.02 | 101.16 | 125.56 | 144.50 | 211.44 | 5.06 | 90 | 28 | 71.25 | 91.00 | 110.00 | 152.00 | 0.00 | **<0.0001** |
| LYM | 79 | 20.70 | 29.10 | 33.40 | 38.30 | 47.80 | 0.00 | 90 | 4 | 13.13 | 19.75 | 26.45 | 52.70 | 0.00 | **<0.0001** |
| MCHC | 79 | 31.80 | 33.40 | 33.80 | 34.10 | 35.00 | 0.00 | 82 | 31 | 32.83 | 33.40 | 34.05 | 36.40 | 8.89 | **0.0099** |
| MCV | 79 | 78.00 | 88.50 | 91.00 | 94.50 | 100.00 | 0.00 | 90 | 80 | 86.70 | 89.75 | 92.10 | 106.70 | 0.00 | **0.0376** |
| MON | 79 | 3.80 | 5.40 | 6.40 | 7.45 | 12.00 | 0.00 | 90 | 2 | 6.03 | 8.40 | 11.13 | 29.80 | 0.00 | **0.0001** |
| MOSM | 0 |  |  |  |  |  | 100.00 | 35 | 238 | 283.85 | 287.10 | 293.10 | 305.90 | 61.11 |  |
| MPV | 79 | 6.80 | 7.70 | 7.90 | 8.60 | 9.60 | 0.00 | 82 | 9 | 9.93 | 10.40 | 11.19 | 13.85 | 8.89 | **<0.0001** |
| NE | 79 | 0.00 | 215.05 | 453.10 | 598.95 | 2045.00 | 0.00 | 90 | 344 | 962.50 | 1153.00 | 1443.25 | 4165.00 | 0.00 | **<0.0001** |
| NEU | 79 | 42.70 | 51.85 | 55.70 | 60.55 | 72.80 | 0.00 | 90 | 35 | 60.08 | 70.80 | 79.40 | 89.20 | 0.00 | **<0.0001** |
| NSE | 79 | 4.59 | 6.81 | 8.58 | 10.88 | 38.38 | 0.00 | 90 | 4 | 10.38 | 14.10 | 19.66 | 64.83 | 0.00 | **<0.0001** |
| NTPROBNP | 0 |  |  |  |  |  | 100.00 | 76 | 8 | 63.83 | 127.50 | 332.85 | 4739.30 | 15.56 |  |
| PCO2 | 0 |  |  |  |  |  | 100.00 | 35 | 30 | 36.85 | 41.70 | 44.00 | 60.40 | 61.11 |  |
| PCT | 0 |  |  |  |  |  | 100.00 | 90 | 0 | 0.03 | 0.05 | 0.08 | 0.48 | 0.00 |  |
| PH | 0 |  |  |  |  |  | 100.00 | 35 | 7 | 7.41 | 7.43 | 7.48 | 7.55 | 61.11 |  |
| PLT | 79 | 159.00 | 213.00 | 243.00 | 278.50 | 367.00 | 0.00 | 90 | 2 | 152.50 | 203.00 | 296.25 | 654.00 | 0.00 | **0.0116** |
| PO2 | 0 |  |  |  |  |  | 100.00 | 35 | 15 | 21.85 | 32.70 | 60.60 | 121.00 | 61.11 |  |
| POTASSIUM | 0 |  |  |  |  |  | 100.00 | 90 | 3 | 3.90 | 4.21 | 4.55 | 157.12 | 0.00 |  |
| PT | 0 |  |  |  |  |  | 100.00 | 87 | 10 | 11.85 | 12.70 | 13.60 | 50.90 | 3.33 |  |
| RBC | 79 | 3.99 | 4.58 | 4.83 | 5.12 | 5.68 | 0.00 | 90 | 3 | 4.17 | 4.56 | 4.85 | 6.22 | 0.00 | **<0.0001** |
| RDWCV | 79 | 36.00 | 40.00 | 42.00 | 43.00 | 52.00 | 0.00 | 82 | 34 | 40.28 | 42.58 | 45.30 | 65.65 | 8.89 | 0.1460 |
| RDWSD | 79 | 11.50 | 12.30 | 13.00 | 13.90 | 17.50 | 0.00 | 82 | 12 | 12.46 | 12.88 | 13.60 | 17.88 | 8.89 | 0.6371 |
| S100B | 79 | 0.00 | 0.00 | 0.00 | 0.00 | 11270.00 | 0.00 | 90 | 556 | 765.03 | 894.04 | 1062.38 | 8095.65 | 0.00 | **<0.0001** |
| SO2 | 0 |  |  |  |  |  | 100.00 | 35 | 17 | 35.70 | 64.30 | 91.10 | 99.20 | 61.11 |  |
| SODIUM | 0 |  |  |  |  |  | 100.00 | 89 | 116 | 137.00 | 139.00 | 142.00 | 152.00 | 1.11 |  |
| STAND_BICARBONATE | 0 |  |  |  |  |  | 100.00 | 35 | 24 | 25.15 | 26.10 | 27.95 | 33.10 | 61.11 |  |
| TCHOL | 79 | 149.00 | 189.50 | 215.00 | 238.00 | 300.00 | 0.00 | 90 | 54 | 131.50 | 152.50 | 183.75 | 245.00 | 0.00 | **<0.0001** |
| TG | 79 | 47.00 | 136.38 | 173.00 | 234.00 | 1350.00 | 0.00 | 90 | 46 | 91.50 | 118.00 | 155.50 | 303.00 | 0.00 | **<0.0001** |
| TPROT | 79 | 6.40 | 7.10 | 7.40 | 7.70 | 213.00 | 0.00 | 31 | 5 | 5.90 | 6.10 | 6.50 | 7.60 | 65.56 | **<0.0001** |
| TSH | 0 |  |  |  |  |  | 100.00 | 90 | 0 | 0.55 | 0.84 | 1.23 | 4.19 | 0.00 |  |
| UREA | 0 |  |  |  |  |  | 100.00 | 90 | 10 | 27.00 | 32.00 | 40.00 | 170.00 | 0.00 |  |
| URICACID | 0 |  |  |  |  |  | 100.00 | 89 | 2 | 3.50 | 4.40 | 5.10 | 12.50 | 1.11 |  |
| WBC | 79 | 3.80 | 5.40 | 6.50 | 7.40 | 10.10 | 0.00 | 85 | 2 | 3.84 | 5.38 | 6.97 | 20.69 | 5.56 | **0.0006** |
| **Qualitative features** | | | | | |  |  |  |  |  |  |  |  |  |  |
| **feature** | **category** | **group** | **n** | **%** | **p** |  |  |  |  |  |  |  |  |  |  |
| s100b positive | no | control | 77 | 97.5 | **<0.0001** |  |  |  |  |  |  |  |  |  |  |
| s100b positive | no | case | 2 | 2.5 |  |  |  |  |  |  |  |  |  |  |  |
| s100b positive | yes | control | 0 | 0 |  |  |  |  |  |  |  |  |  |  |  |
| s100b positive | yes | case | 90 | 100 |  |  |  |  |  |  |  |  |  |  |  |
| sexmale | no | control | 24 | 30.4 | 0.5830 |  |  |  |  |  |  |  |  |  |  |
| sexmale | no | case | 32 | 35.6 |  |  |  |  |  |  |  |  |  |  |  |
| sexmale | yes | control | 55 | 69.6 |  |  |  |  |  |  |  |  |  |  |  |
| sexmale | yes | case | 58 | 64.4 |  |  |  |  |  |  |  |  |  |  |  |
